# Injectable, macroporous scaffolds for delivery of therapeutic genes to the injured spinal cord

**DOI:** 10.1063/5.0035291

**Published:** 2021-03-09

**Authors:** Arshia Ehsanipour, Mayilone Sathialingam, Laila M. Rad, Joseph de Rutte, Rebecca D. Bierman, Jesse Liang, Weikun Xiao, Dino Di Carlo, Stephanie K. Seidlits

**Affiliations:** 1 Department of Bioengineering, University of California, Los Angeles, California 90095, USA; 2 California NanoSystems Institute (CNSI), University of California, Los Angeles, California 90095, USA; 3 Jonsson Comprehensive Cancer Center, University of California, Los Angeles, California 90095, USA; 4 Broad Stem Cell Research Center, University of California, Los Angeles, California 90095, USA; 5 Brain Research Institute, University of California, Los Angeles, California 90095, USA

## Abstract

Biomaterials are being developed as therapeutics for spinal cord injury (SCI) that can stabilize and bridge acute lesions and mediate the delivery of transgenes, providing a localized and sustained reservoir of regenerative factors. For clinical use, direct injection of biomaterial scaffolds is preferred to enable conformation to unique lesions and minimize tissue damage. While an interconnected network of cell-sized macropores is necessary for rapid host cell infiltration into—and thus integration of host tissue with—implanted scaffolds, injectable biomaterials have generally suffered from a lack of control over the macrostructure. As genetic vectors have short lifetimes *in vivo*, rapid host cell infiltration into scaffolds is a prerequisite for efficient biomaterial-mediated delivery of transgenes. We present scaffolds that can be injected and assembled *in situ* from hyaluronic acid (HA)-based, spherical microparticles to form scaffolds with a network of macropores (∼10 *μ*m). The results demonstrate that addition of regularly sized macropores to traditional hydrogel scaffolds, which have nanopores (∼10 nm), significantly increases the expression of locally delivered transgene to the spinal cord after a thoracic injury. Maximal cell and axon infiltration into scaffolds was observed in scaffolds with more regularly sized macropores. The delivery of lentiviral vectors encoding the brain-derived neurotrophic factor (BDNF), but not neurotrophin-3, from these scaffolds further increased total numbers and myelination of infiltrating axons. Modest improvements to the hindlimb function were observed with BDNF delivery. The results demonstrate the utility of macroporous and injectable HA scaffolds as a platform for localized gene therapies after SCI.

## I. INTRODUCTION

As of 2020, spinal cord injury (SCI) is estimated to have an average annual incidence of 54 cases per million people in the United States (roughly 17 800 annually), and the consequences to those affected are tremendous, often resulting in mild to severe paraplegia, bladder dysfunction, sexual dysfunction, and/or spasticity.^1^ The estimated lifetime cost of treating each patient with SCI in the U.S. ranges from $1 × 10^6^ to $5 × 10^6^, depending on severity and age at injury.^1^ Development of novel therapies that improve tissue sparing and/or induce repair after SCI could alleviate these personal and financial costs for patients in the future.^2^

A substantial portion of neurological damage caused by SCI has been attributed to the secondary injury response that occurs after the primary traumatic insult, where an ischemic and inflammatory microenvironment propagates cell death, axon retraction, and axon demyelination.^3–5^ Immediately after SCI, immune cells, which include macrophages and neutrophils from the peripheral blood supply and microglia from surrounding tissue, infiltrate the lesion. Activated immune cells produce reactive oxygen species and cytokines that cause extensive death of cells required for the neurological function, in particular conducting neurons and myelinating oligodendrocytes.^3^ While some level of an immune response is necessary to initiate wound healing, an overactive response in the acute phase of SCI and persistence of these inflammatory cells in the chronic phase represent substantial barriers to regeneration.^6^

The chronic pathology of SCI is characterized by the formation of dense scar tissue, typically surrounding a cystic cavity.^3,7^ While some degree of scar formation can prevent the injury from spreading and further cell death,^8^ several components of this scar tissue likely inhibit the ability of regenerating axons to bridge SCI lesions.^3,9,10^ Taken together, previous reports suggest that in order to support tissue regeneration after SCI, the local microenvironment should attenuate, but not eliminate, the immune response and scar formation while actively promoting neuronal survival and axonal growth.

Biomaterial scaffolds can improve SCI outcomes through modulating the inflammatory response to reduce or avoid formation of a cystic cavity walled off by a dense scar and providing a scaffolding for potentially reparative cells to infiltrate the lesion site.^11–13^ In acute SCI, scaffolds may also help to stabilize injured tissue.^14^ Scaffolds that approximate the mechanical properties and water content of native spinal cord tissue have been found to provide this stability, reduce inflammation, and integrate with host tissue.^12,13,15,16^ Hydrogel materials are seemingly ideal for this application, given their relatively high-water content and soft tissue-like mechanics.^17^ Additionally, the ability to deliver hydrogel materials via injection so that scaffolds form *in situ* in the spinal cord makes hydrogels attractive candidates. As the majority of clinical cases of SCI involve incomplete injuries,^18^ administration of biomaterial-based therapies directly into the spinal cord via injection to avoid unintentional damage to spared axonal tracts is desirable.

Despite their advantages, a major limitation to traditional hydrogels is their nanoscale porosity, which restricts infiltration of host cells and axons into lesion sites after implantation.^12^ In contrast, cell-scale macropores can improve host integration by providing a geometric template for infiltrating cells, axons, and vasculature.^19–22^ Incorporation of cell-scale (on the order of 10–100 *μ*m diameter) macropores into scaffolds improves host-cell integration and functional outcomes after SCI in rodents.^23^ However, macroporous scaffolds typically cannot be delivered by injection and instead must be pre-fabricated, cut to fit each unique defect, and positioned carefully within the spinal cord.^23,24^ Although recent work has attempted to tackle this issue using magnetic resonance images as a guide to custom-fabricate scaffolds for an individual patient, this approach is relatively low-throughput and requires specialized analysis and equipment.^25^ To address these issues, we investigated whether a hydrogel-based scaffold that can be injected and forms a macroporous architecture *in situ* can increase host tissue integration and functional outcomes after SCI.

Here, we employed hyaluronic acid (HA), a major structural and bioactive component of the spinal cord extracellular matrix (ECM), as a base material for scaffolds. HA biomaterials have a long history of clinical use for ophthalmic and orthopedic applications.^26,27^ While previous studies have demonstrated that HA biomaterials can reduce inflammation and scarring while promoting angiogenesis after SCI in rodents, these studies reported poor infiltration of cells and axons and integration with host tissue.^12,28^ Here, we demonstrate that addition of a network of regularly structured, cell-scale macropores within HA-based scaffolds substantially increases infiltration of host cells and axons after SCI in a mouse model.

Biomaterial scaffolds can also act as local reservoirs of bioactive factors (e.g., through gene delivery) that can promote tissue sparing and regeneration.^29^ Many studies have the explored delivery of various biological factors, including proteins and nucleic acids, to alter the local microenvironment after SCI. Neurotrophic factors that modulate axon growth and neuroplasticity, such as brain-derived neurotrophic factor (BDNF) and neurotrophin-3 (NT3), have been reported to be beneficial after SCI.^30–33^ The delivery of the BDNF, either systemically or intrathecally, has been reported to improve tissue regeneration and functional recovery in animal^30,31^ and human trials.^34^ However, the delivery of growth factors by bolus injection requires supraphysiological doses, which may cause undesired side effects, is expensive, and results in significant variability of factor availability over time.^35–37^ Continuous infusion of growth factors requires the use of implanted intrathecal pumps,^31^ which require more invasive procedures and have been shown to cause observable tissue damage.^38^ Alternatively, the delivery of cells engineered to overexpress proteins, such as BDNF, to the spinal cord has been explored as a strategy to provide a sustained source of beneficial factors that remain localized near the delivery site.^13,39^ However, cell injection into the spinal cord typically results in low cell survival and may require continued administration of immunosuppressive agents.^30^

In preclinical models of SCI, bioengineered scaffolds have been explored for their ability to support the local delivery of proteins^40–45^ or genetic vectors.^46–48^ Transgene delivery, achieved through viral or non-viral vectors, avoids several concerns with protein delivery, including (1) loss of protein bioactivity over time, (2) expense and supply limitations given large amounts of protein required for therapeutic loading, and (3) the need to tailor biomaterial chemistry for each specific protein to be delivered. Genetic vectors have the flexibility of delivering transmembrane proteins,^49^ silencing RNA,^50^ or transcription factors^51^ in addition to secreted proteins.^29,47^ Biomaterial-mediated gene delivery provides an excellent platform for the development of complex, combinatorial therapies, which often have synergistic benefits on functional recovery after SCI.^6,47,48,52^ With viral carriers, the same packaging vector can be used to deliver multiple therapeutic genes without the need to re-engineer biomaterial chemistry to enable interactions with each individual therapeutic molecule. Viral vectors have shown particular promise for the delivery of therapeutic transgenes, given their substantially higher transduction efficiency compared to non-viral vectors.^53^ Here, we investigated lentiviral vectors for delivery, which can infect both dividing and non-dividing cells, have broad tropism, integrate into the host genome to yield long-term effects, and are relatively straightforward to produce.

Transduction efficiency from biomaterials depends on the ability of cells to infiltrate the biomaterial and interact with vectors within the first day or two after implantation before vectors lose their activity.^19^ While in theory cells could encounter and be transduced by vectors diffusing out of the scaffold, infiltration of cells into the scaffold has been found to dominate transgene uptake, most likely due to the large size of lentiviral vectors relative to proteins and small molecules and the short half-life of lentivirus *in vivo* (estimated to be 24–48 h).^23,54–56^ This dependence on rapid cell infiltration necessitates the presence of a macroporous architecture in scaffolds. Studies by our group and others have confirmed that scaffold porosity dictates the efficiency of biomaterial-mediated transgene delivery.^19,54^ Previous studies have shown that lentiviral vectors, including those encoding BDNF and NT3, can be efficiently delivered to the spinal cord after injury from macroporous biomaterials and that transgene expression persists at initial levels for at least 8 weeks in rodent models.^29,47^

To create injectable scaffolds with macroporous architectures, we utilized a previously developed strategy in which spherical hydrogel particles can be injected and then tether to each other *in situ* so that the void space around the particles leaves an interconnected network of macropores.^54,57,58^ These scaffolds can be created using hydrogel particles with wide (polydisperse) or narrow (monodisperse) size distributions. Packing densities of polydisperse and monodisperse particles have been previously studied with a focus on solid state systems where Lochmann *et al.* found that the theoretical packing density of monodisperse particles, 64%, increases to 74% if particle distributions span an order of magnitude and further increases with greater variation.^59^ Since void space is defined by unpacked space, it follows that polydisperse particles provide less available space for infiltrating cells.

Here, we investigated how the presence of a macroporous architecture, and the structure of this architecture, affects inflammation, scar formation, infiltration of host cells and axons, and functional recovery after thoracic-level SCI in a mouse model. We compared scaffolds with the same HA-based chemical composition and with varying structures: two macroporous architectures, created from annealing hydrogel particles with monodisperse or polydisperse diameters, and traditional, nanoporous (NP) hydrogels. As cell infiltration is a key determinant of successful gene delivery from scaffolds,^19^ the effects of pore architecture on the efficiency of transgene delivery from lentivirus-laden scaffolds were also investigated. Finally, we investigated the therapeutic effects of delivering lentiviral vectors encoding neurotrophic factors from biomaterial scaffolds.

## II. RESULTS

Annealed microparticle scaffolds (pHA-MP and mHA-MP) were developed to provide micrometer-scale pores within an *in situ* crosslinked scaffold for the delivery of lentiviral particles and regeneration of spinal cord tissue [Figs. S1(A) and S1(B)]. For pHA-MP scaffolds, microparticle diameters measured 42 ± 24 *μ*m and, for mHA-MP scaffolds, 55 ± 4 *μ*m [Fig. S1(C)]. A lack of diffusion of high molecular weight FITC-dextran (Stokes radius, ∼15 nm^60^) confirmed that NP-HA scaffolds were difficult for large solutes to penetrate [Fig. S1(D)], whereas diffusion was ample through the void spaces formed by packed microparticles for both pHA-MP and mHA-MP scaffolds [Figs. S1(E) and S1(F)]. Scaffold void spaces were larger and more consistent in mHA-MP scaffolds (47% ± 1%) than in pHA-MP scaffolds (33% ± 8%) [Fig. S1(G)].

**FIG. 1.**
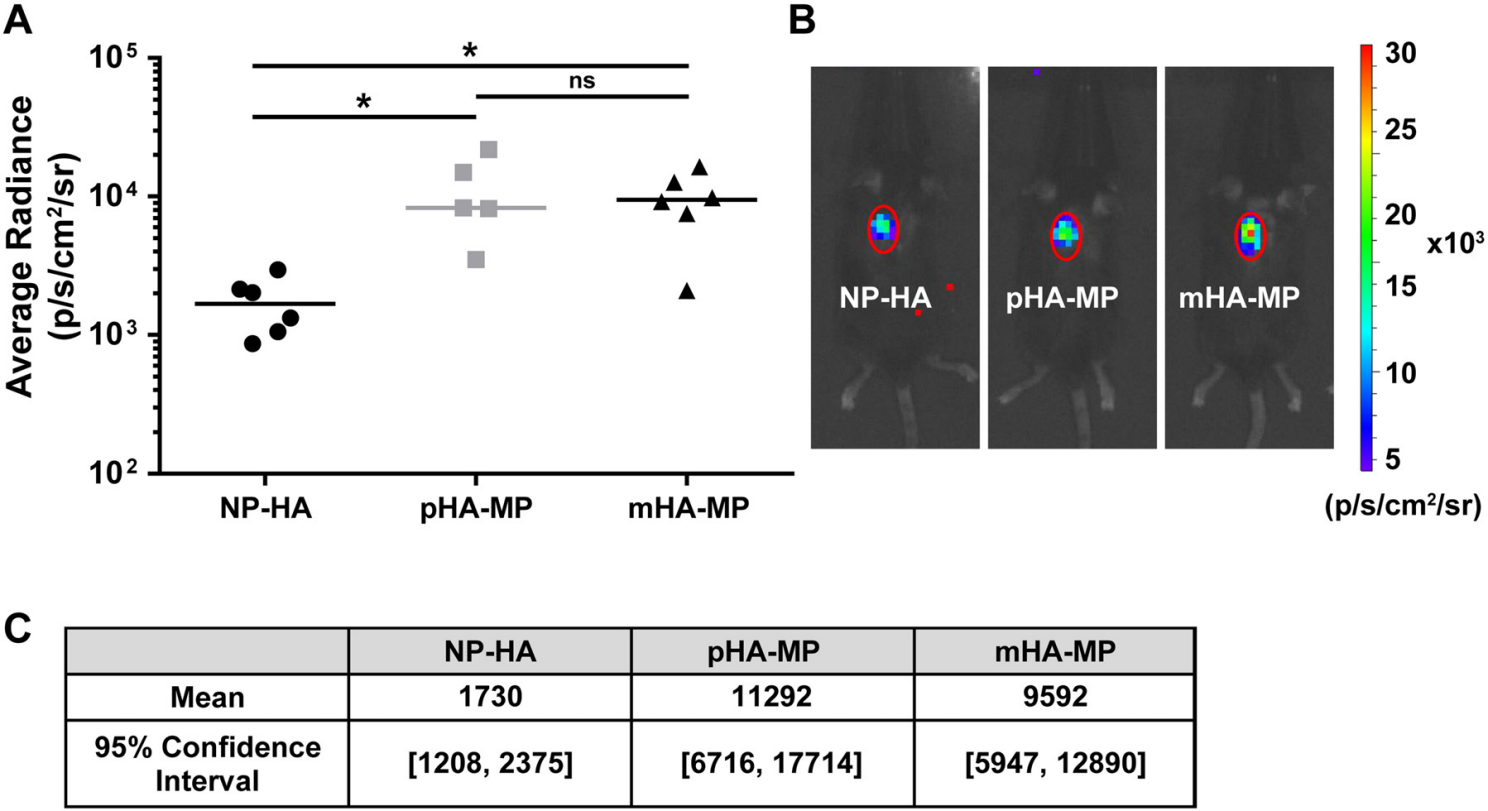
Delivery of FLuc-encoding lentivirus to the injured spinal cord. Both pHA-MP and mHA-MP scaffolds exhibited significantly greater integrated bioluminescence intensity than NP-HA scaffolds [(a) and (b)] 2 weeks post-injection. There was no significant difference between pHA-MP and mHA-MP scaffolds. Bootstrap analysis with 10 000 iterations agreed with statistical testing (c) (^*^p <* *0.05, Kruskal-Wallis test with Dunn's multiple comparisons test, n = 5–6).

**FIG. 2.**
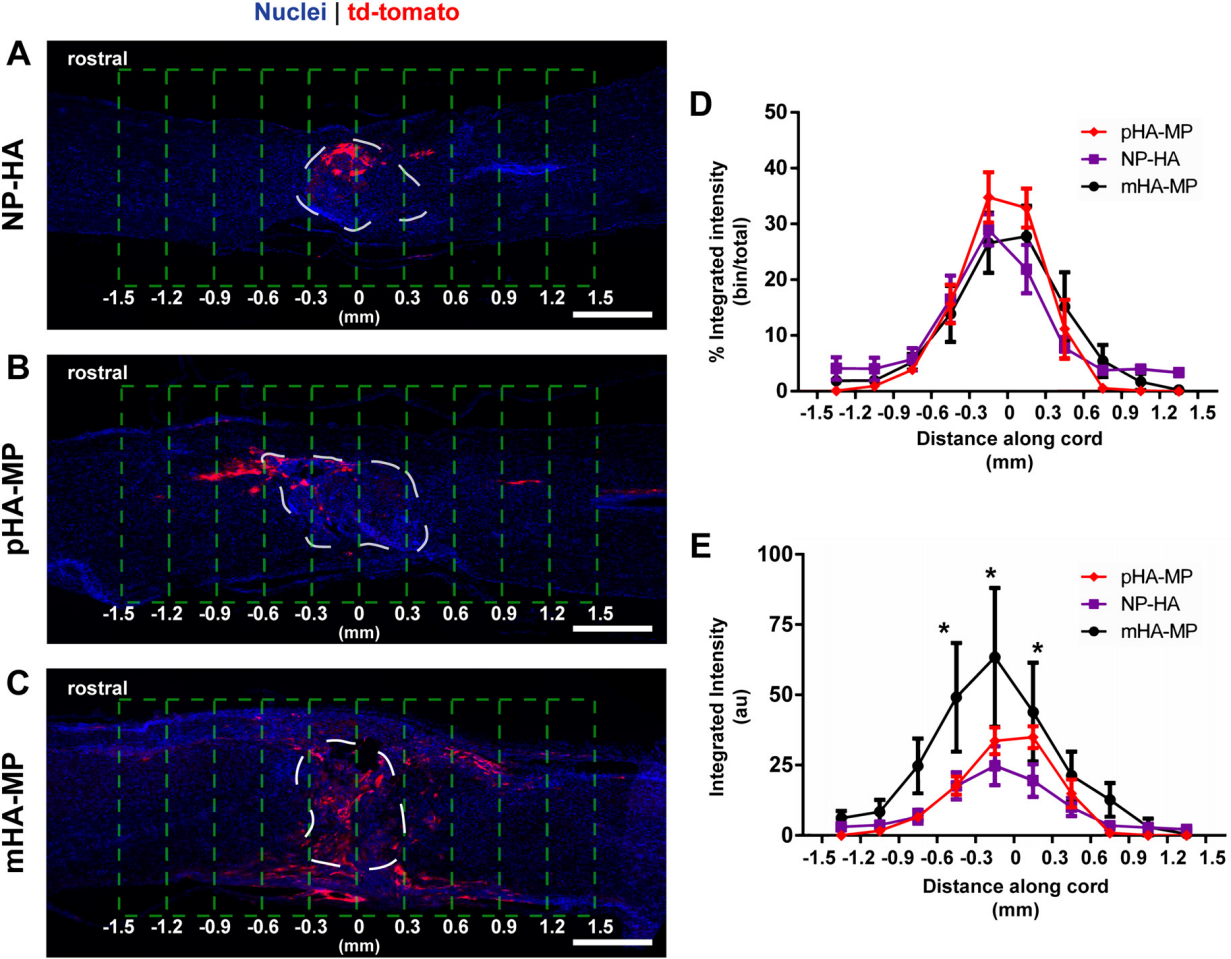
Expression of td-Tomato transgene was present throughout spinal cords 8 weeks post-injury but localized near the site of injection. Td-Tomato transgene is visible near lesion sites where lentivirus-loaded NP-HA (a), pHA-MP (b), and mHA-MP (c) scaffolds were injected. White dashed lines indicate the scaffold region, and green dashed lines indicate the areas used for quantification. There were no significant differences in the distribution of transgene expression within 1.5 mm of lesions, as measured by comparing integrated fluorescence intensity in 300 *μ*m segments normalized to the total integrated fluorescence intensity within 1.5 mm of the scaffold center (d). However, mHA-MP scaffolds had a greater raw td-Tomato fluorescence intensity signal within 300 *μ*m of the scaffold center in either direction (E) (^*^p <* *0.05, two-way repeated measures ANOVA, Tukey test, n = 3–4 animals, scale bars = 500 *μ*m).

To evaluate how the porous architecture of scaffolds affected lentiviral delivery to lesions in acute SCI, we delivered lentivirus encoding overexpression of FLuc within NP-HA, pHA-MP, and mHA-MP scaffolds. Scaffolds with cell-scale macroporosity (pHA-MP and mHA-MP) resulted in significantly greater bioluminescence than nanoporous scaffolds (NP-HA), indicating greater expression levels of FLuc transgene by host cells (Fig. 1). A 2-week time point was selected as previous reports have found that expression of scaffold-delivered transgenes in the injured, rodent spinal cord peaks at 2 weeks and continues at a relatively constant level for at least 8 weeks.^23,46,47^ To further examine transgene expression throughout scaffolds, lentivirus encoding the reporter gene td-Tomato was delivered from each scaffold type after SCI. Td-Tomato transgene expression was primarily localized at the site of injection in all scaffold types, but expression was observed as far as 1.5 mm from the injection site [Figs. 2(a)–2(c)]. Expressed td-Tomato was quantified in fixed cryosections [Figs. 2(d) and 2(e)]. The normalized fluorescence signal from expressed td-Tomato transgene (tissue region/total signal) was not significantly different among scaffolds, indicating that expression remained localized near all scaffold implants [Fig. 2(d)]. The total fluorescence intensity was also quantified to identify any differences in absolute transgene expression. Consistent with measurements of bioluminescence of FLuc transgene, the fluorescence signal from td-Tomato transgene was greater in mice treated with mHA-MP scaffolds, in particular within 300 *μ*m of scaffold centers [Fig. 2(e)].

To account for the possibility that tissue preparation (i.e., fixation and cryosectioning) might have prevented fluorescence of td-Tomato, we compared the area covered by fluorescence from unamplified td-Tomato to that in adjacent tissue sections when an antibody is used against td-Tomato, which should detect any potentially bleached td-Tomato [Figs. S2(A)–S2(C)]. Immunostained and non-immunostained td-Tomato fluorescence signals did not cover significantly different tissue areas, with a difference of 10% ± 5% between fluorescently positive areas between immunostained and non-stained signals across all samples [Fig. S2(D)]. Thus, analyses were performed without immunostaining for td-Tomato.

**FIG. 3.**
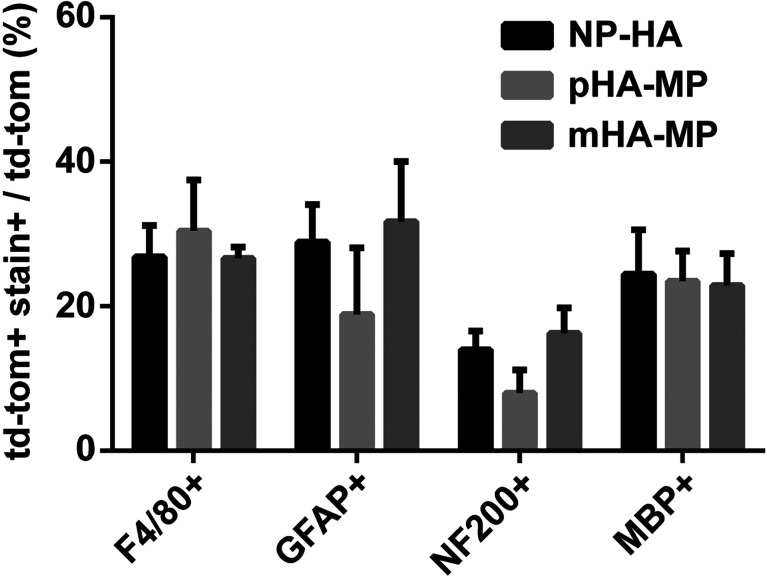
Quantification of td-Tomato transgene co-staining with markers of neural and inflammatory cell types at 8 weeks post-injury. Td-Tomato transgene co-stains with each neural and inflammatory cell type stained. No significant differences were observed between each scaffold condition (*p *<* *0.05, Kruskal-Wallis test with Dunn's multiple comparisons test, n = 3–4).

To identify which cell types were transduced by viral vectors delivered via each scaffold, we used antibodies against markers of astrocytes (GFAP^+^), axons (NF200^+^), myelinating cells (MBP^+^), and immune cells (F4/80^+^, macrophages/microglia). Quantification indicated no differences in the extent of transgene expression among cell types examined, with roughly 25% of transgene being expressed by macrophages/microglia, 25% by astrocytes, 20% by myelinating cells, and 10% with neurons (Fig. 3). While we did not uncover here the identities of the remaining ∼30% of cells expressing transgene, previous reports suggest that these are likely fibroblasts, endothelial cells, or non-myelinating glia.^14,23,61^

**FIG. 4.**
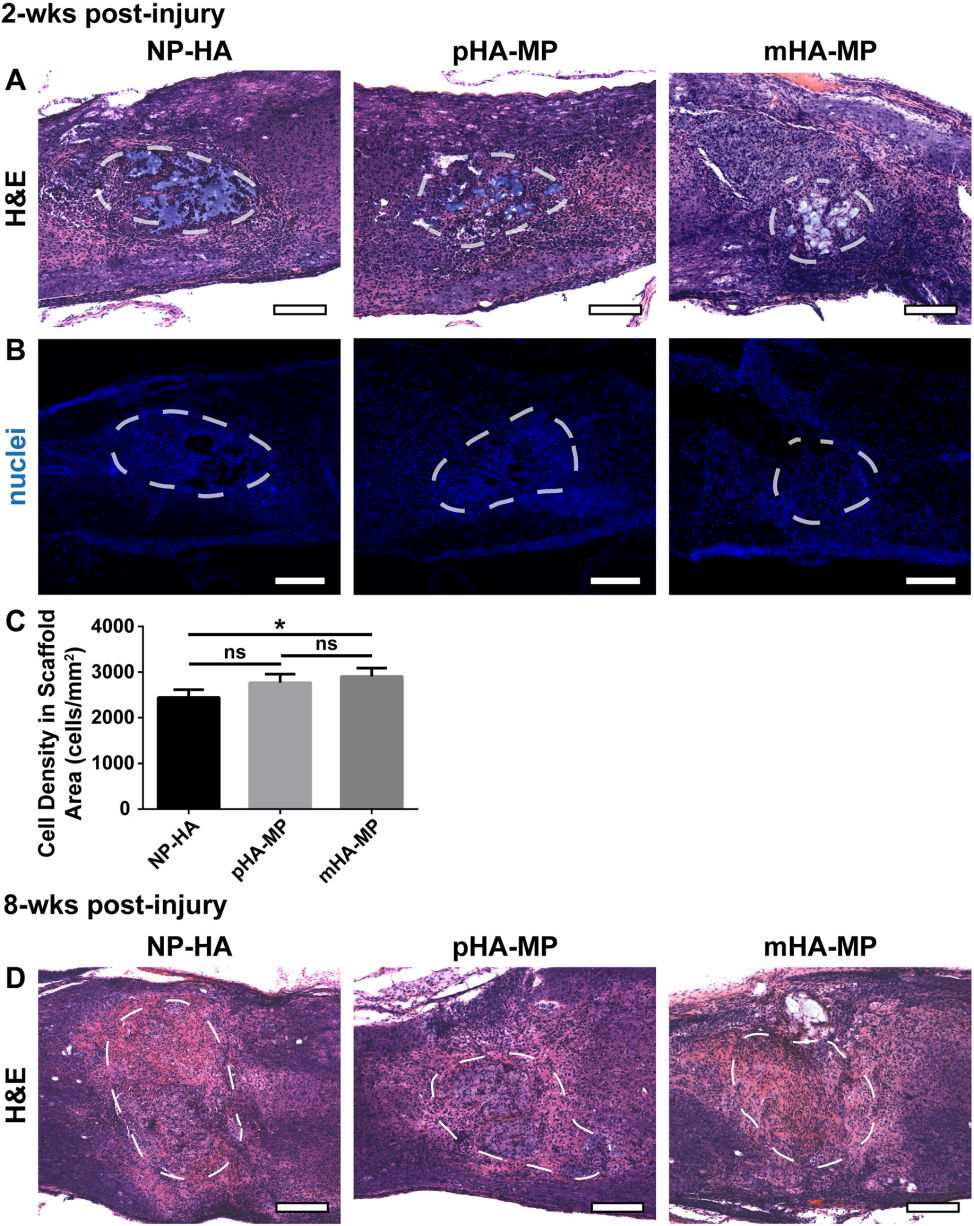
H&E and nuclei staining of spinal cord 2 and 8 weeks post-injury. Scaffolds were clearly identifiable 2 weeks post-injury in H&E stained sections, outlined in white dashed lines (a). Cells infiltrate into each scaffold type, though regions devoid of nuclei were observed, particularly in NP-HA scaffolds (b). Quantification of numbers of nuclei in scaffolds showed that mHA-MP scaffolds had significantly more cell infiltration than NP-HA, but not pHA-MP, scaffolds (c). Scaffolds were still identifiable 8 weeks post-injury in H&E stained sections (d) (^*^p <* *0.05, Kruskal-Wallis test with Dunn's multiple comparisons test, n = 4–5, scale bars = 200 *μ*m).

**FIG. 5.**
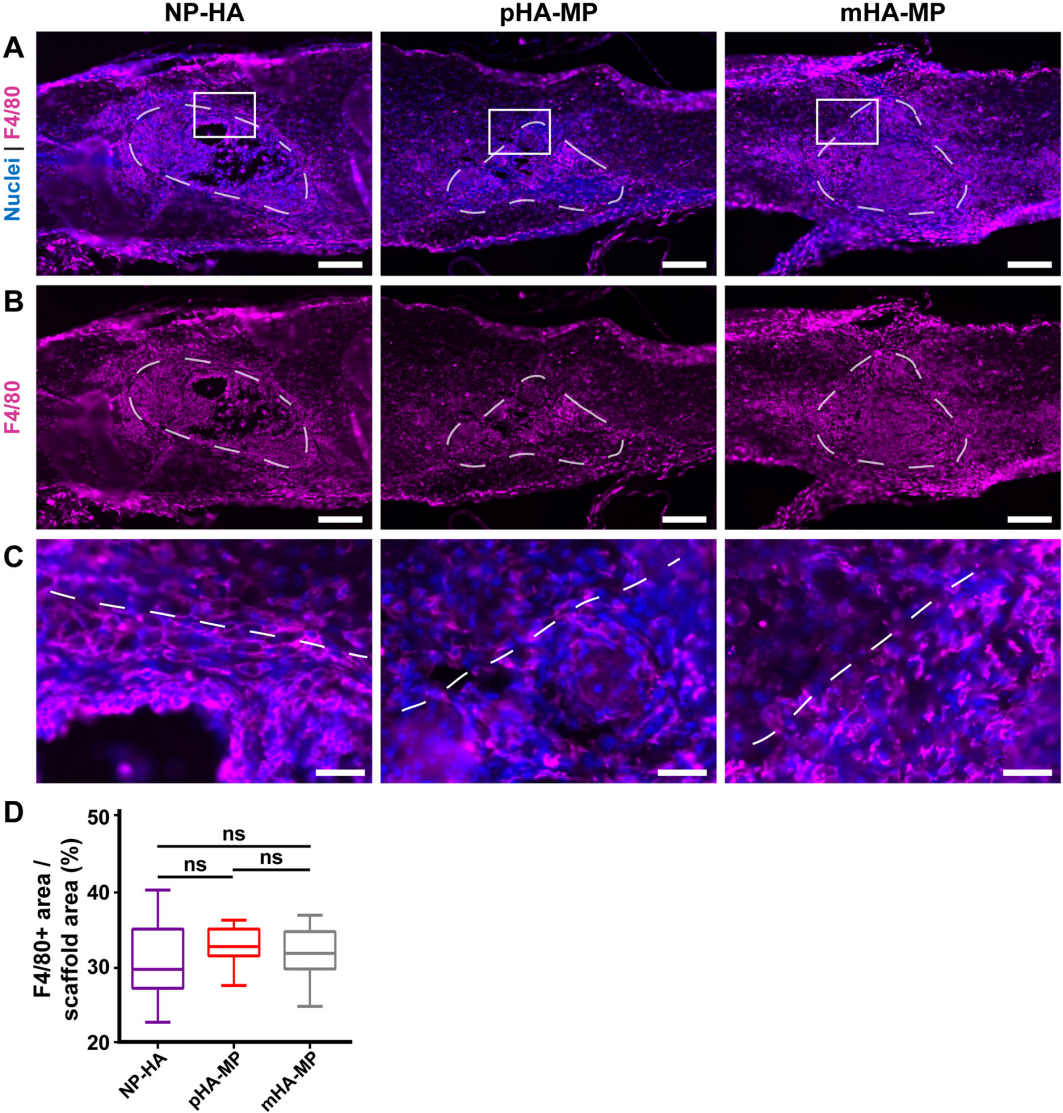
Immunostaining of inflammatory cell types in scaffolds 2 weeks post-injury. F4/80^+^ macrophages/microglia were the most abundant cell type within scaffolds [(a)–(c)], and no significant difference was found between scaffold types (d) {^*^p <* *0.05, Kruskal-Wallis test with Dunn's multiple comparisons test, n = 5–6 animals, scale bars = 200 *μ*m [(a)–(c)], 40 *μ*m (d)}.

**FIG. 6.**
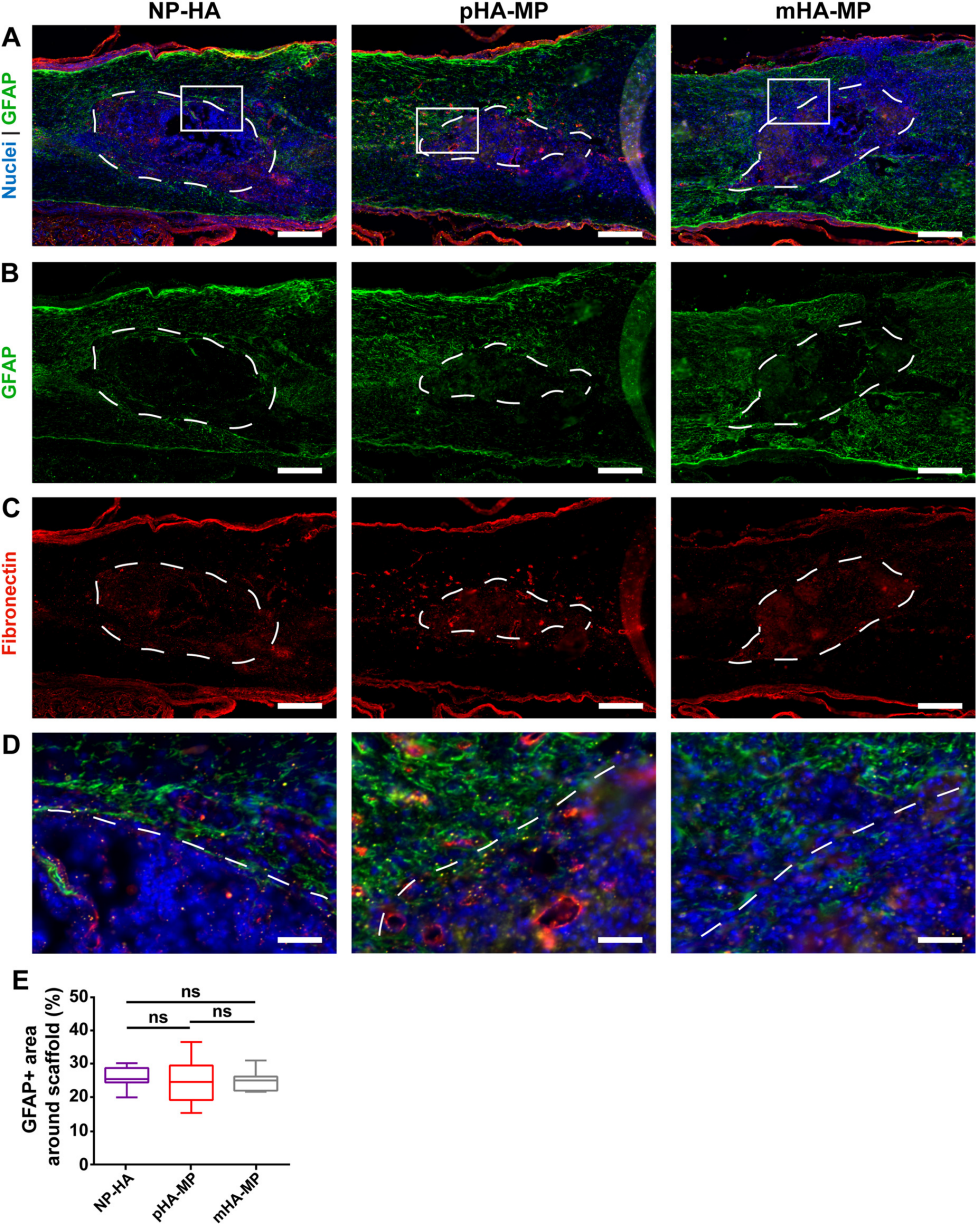
Immunostaining of astrocytic cell types and glial scar in scaffolds 2 weeks post-injury. GFAP^+^ astrocytes are found throughout the spinal cord (a), but denser staining was observed surrounding scaffolds (b), also associated with deposited fibronectin (c), consistent with previous work showing an activated astrocyte barrier. Zoomed insets (d) show scaffolds' borders. No significant differences in the GFAP^+^ area within 200 *μ*m of scaffold borders were observed along scaffold types (e) [^*^p <* *0.05, Kruskal-Wallis test with Dunn's multiple comparisons test, n = 5–6, scale bars = 200 *μ*m (a)–(c), 40 *μ*m (d)].

Tissue integration was assessed from H&E-stained cryosections and cell infiltration quantified by counting Hoechst-stained nuclei in cryosections. Scaffolds were clearly identifiable at the center of the injury site 2 weeks [Figs. 4(a)–4(c)] or 8 -weeks [Fig. 4(d)] post-injury, and lesion pathology was consistent with moderate SCI.^68,78^ While there is a clear variation in lesion areas within each condition, we did not identify any significant differences in injury areas across scaffold conditions (Fig. S3). Regardless of scaffold type, substantial numbers of cells infiltrated scaffolds; however, only NP-HA scaffolds exhibited large (>100 *μ*m across) regions devoid of any nuclei [Fig. 4(b)]. pHA-MP and mHA-MP scaffolds appeared to have fewer regions devoid of cells than NP-HA scaffolds [Fig. 4(b)]. Quantification of infiltrating cells (normalized to the area) 2 weeks after injury found significantly more nuclei present within mHA-MP than NP-HA, but not pHA-MP, scaffolds [Figs. 4(c) and 4(d)]. Two weeks after SCI, there were no apparent differences in numbers of macrophages/microglia (F4/80^+^) within scaffolds (Fig. 5) or the area covered by reactive astrocytes (GFAP^+^) immediately surrounding scaffold implants (Fig. 6).

**FIG. 7.**
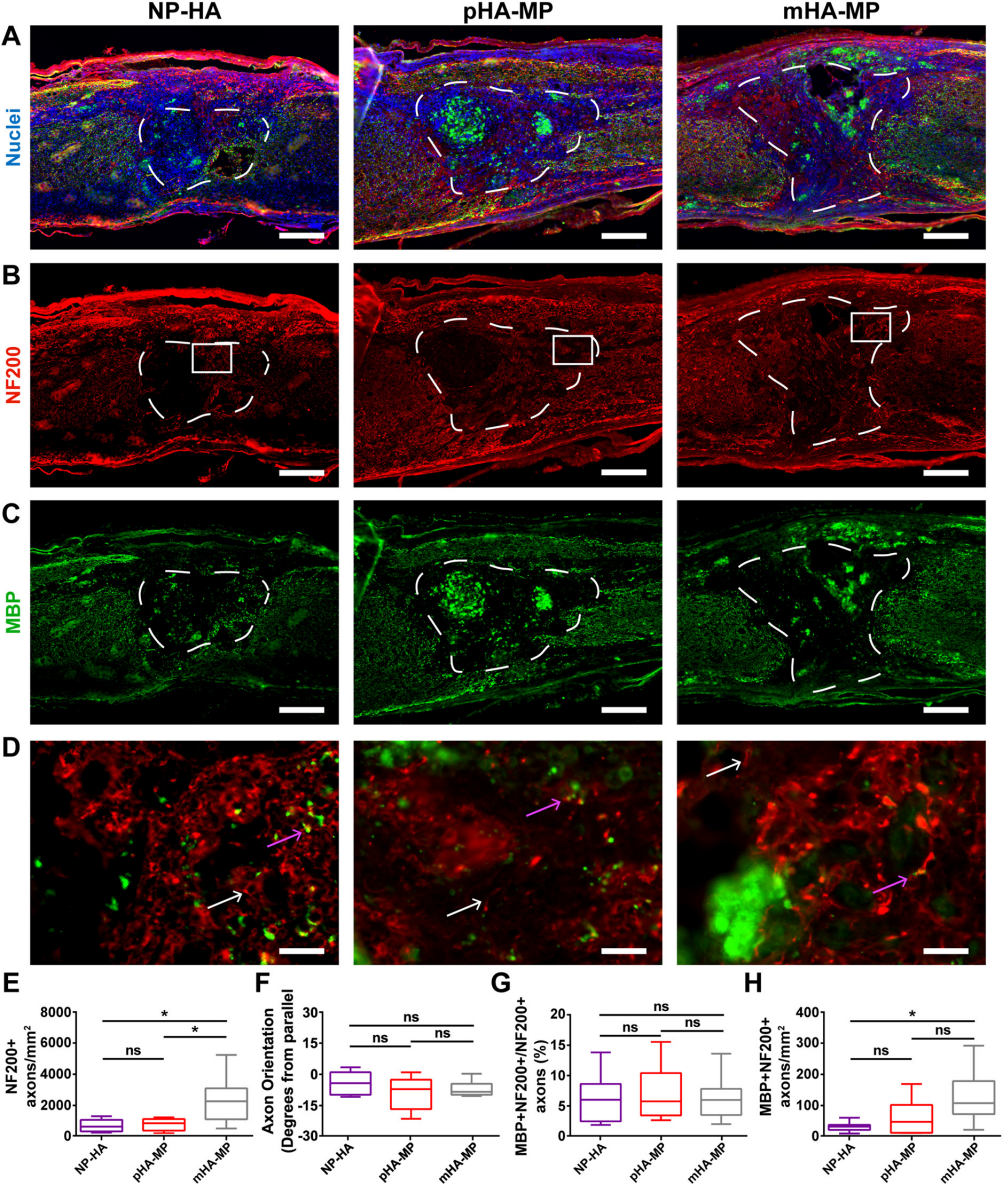
Immunostaining of myelinated axons in scaffolds 8 weeks post-injury. Spinal cords injected with NP-HA, pHA-MP, and mHA-MP scaffolds (a) showed clear scaffold boundaries that NF200^+^ axons (b) and MBP^+^ oligodendrocytes (c) appeared to cross. Zoomed-in images of the areas indicated by white boxes in (b) are shown in (d). White arrows indicate NF200^+^/MBP^−^ axons, while pink arrows indicate NF200^+^/MBP^+^ axons. NF200^+^ axons had significantly greater densities in mHA-MP scaffolds than NP-HA or pHA-MP (e). The net orientation of axons was near parallel, relative to the longitudinal axis of the spinal cord, in each condition, with no significant differences among conditions (f). There was no difference in the percentage of axons that were myelinated (g). There was greater myelinated axon density in mHA-MP scaffolds than in NP-HA, but not pHA-MP, scaffolds (h) [^*^p <* *0.05, Kruskal-Wallis test with Dunn's multiple comparisons test, n = 5–6, scale bars = 200 *μ*m for (a)–(c) and 20 *μ*m for (d), white arrows = unmyelinated axon and pink arrows = myelinated axon].

Significantly, more axons (NF200^+^) were found to infiltrate scaffolds with a highly uniform, macroporous architecture, mHA-MP, compared to NP-HA and pHA-MP scaffolds [Figs. 7(a)–7(e)]. This increase in axon ingrowth 8 weeks after SCI is consistent with the increase in cell infiltration observed within the mHA-MP scaffolds 2 weeks after SCI, suggesting that uniform macroporosity facilitated migration of both cells and axons into scaffolds. The orientation of axons that had infiltrated scaffolds 8 weeks after SCI was found to be closely aligned with the longitudinal place for all scaffold and transgene conditions with ±10° difference between the net angles of NF200+ objects from the longitudinal axis of the spinal cord [Fig. 7(f)]. While there were no differences in the proportion of axons with myelin (double positive for MBP and NF200 divided by total positive for NF200) [Fig. 7(g)], there was a significant increase in the total density of myelinated axons in mHA-MP scaffolds relative to NP-HA and pHA-MP [Fig. 7(h)].

Next, we evaluated the scaffold-mediated delivery of lentiviral vectors encoding for the overexpression of the neurotrophic factors BDNF or NT3. Prior to *in vivo* studies, each batch of lentiviral vectors was evaluated for potency by first infecting HEK cells and then applying the HEK cell-conditioned medium, which should now contain BDNF or NT3, to DRGs (Fig. S4). The results confirmed the production of the appropriate growth factor by infected HEK cells and the ability of secreted protein to promote survival and elongation of DRG neurites in culture.

**FIG. 8.**
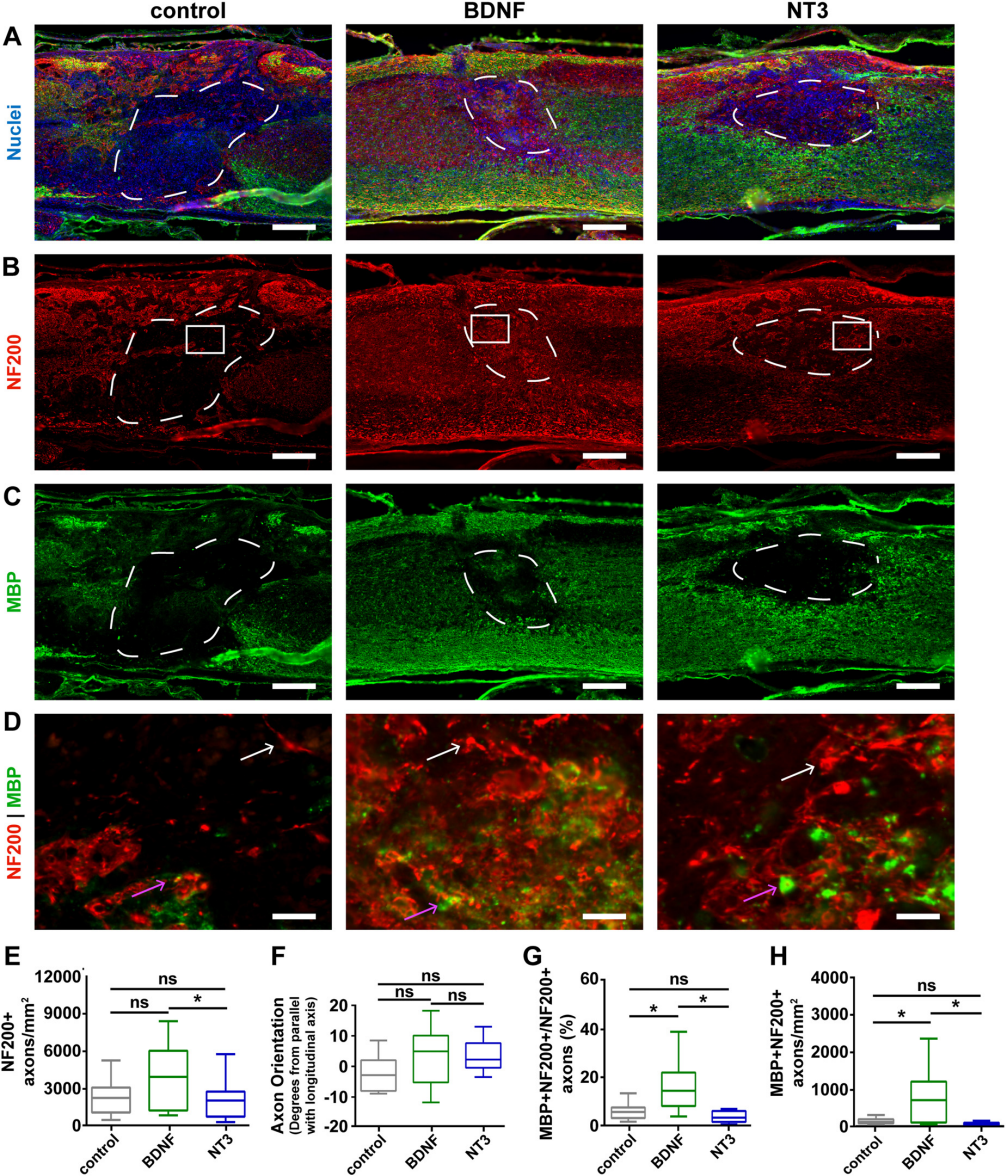
Quantification of myelinated axons after the delivery of neurotrophic factor-encoding vectors 8 weeks post-injury. mHA-MP scaffolds were delivered after injury containing BDNF, NT3, or control FLuc-encoding vectors (a) and showed NF200^+^ axons (b) and MBP^+^ oligodendrocytes (c) within the scaffolds. Zoomed-in images of the areas indicated by white boxes in (b) are shown in (d). White arrows indicate NF200^+^/MBP^-^ axons, while pink arrows indicate NF200^+^/MBP^+^ axons. The delivery of vectors encoding for the BDNF increased the density of axons (NF200^+^) and myelinated axons (NF200^+^/MBP^+^). The axonal density was significantly higher with the delivery of the BDNF relative to NT3 (*p *<* *0.1) but not FLuc control (e). The net orientation of axons was near parallel, relative to the longitudinal axis of the spinal cord, in each condition, with no significant differences among conditions (f). BDNF delivery led to increased proportion of myelinated axons (g) and densities of myelinated axons (h) relative to both NT3 and control ([*p *<* *0.05, Kruskal-Wallis test with Dunn's multiple comparisons test, n = 5–6, scale bars = 200 *μ*m for (a)–(c) and 20 *μ*m for (d), white arrows = unmyelinated axon and pink arrows = myelinated axon].

mHA-MP scaffolds were used for *in vivo* studies of gene delivery as they most effectively increased cell infiltration (Fig. 4), transgene expression [Figs. 1 and 2(e)], and axon ingrowth [Fig. 7(e)]. High densities of cells were visible 8 weeks post-injury in H&E-stained sections [Fig. 4(d)]. The delivery of either BDNF or NT3 transgenes from mHA-MP scaffolds had substantial effects on axon infiltration and myelination 8 weeks after SCI (Fig. 8). The density of NF200^+^ neurons in scaffolds was roughly twofold higher with the delivery of BDNF transgene than the delivery of NT3 (however, this difference was not statistically significant) or reporter (FLuc or td-Tomato control vectors) transgenes (this difference was statistically significant) [Figs. 8(a)–8(e)]. There were no significant differences between the orientations of axons that had infiltrated the scaffolds, with ±10° difference between the net angles of NF200+ objects from the longitudinal axis of the spinal cord [Fig. 8(f)]. The delivery of BDNF transgene led to significantly greater proportions of axons that were myelinated (NF200^+^ and MBP^+^) and total densities of myelinated axons (NF200^+^ and MBP^+^) than the delivery of NT3 or control transgenes [Figs. 8(g) and 8(h)]. There were few infiltrating Schwann cells (P0^+^), which could contribute to regenerated myelin sheaths, in all conditions, but there appeared relatively more Schwann cells in the NT3 condition (Fig. S5).

**FIG. 9.**
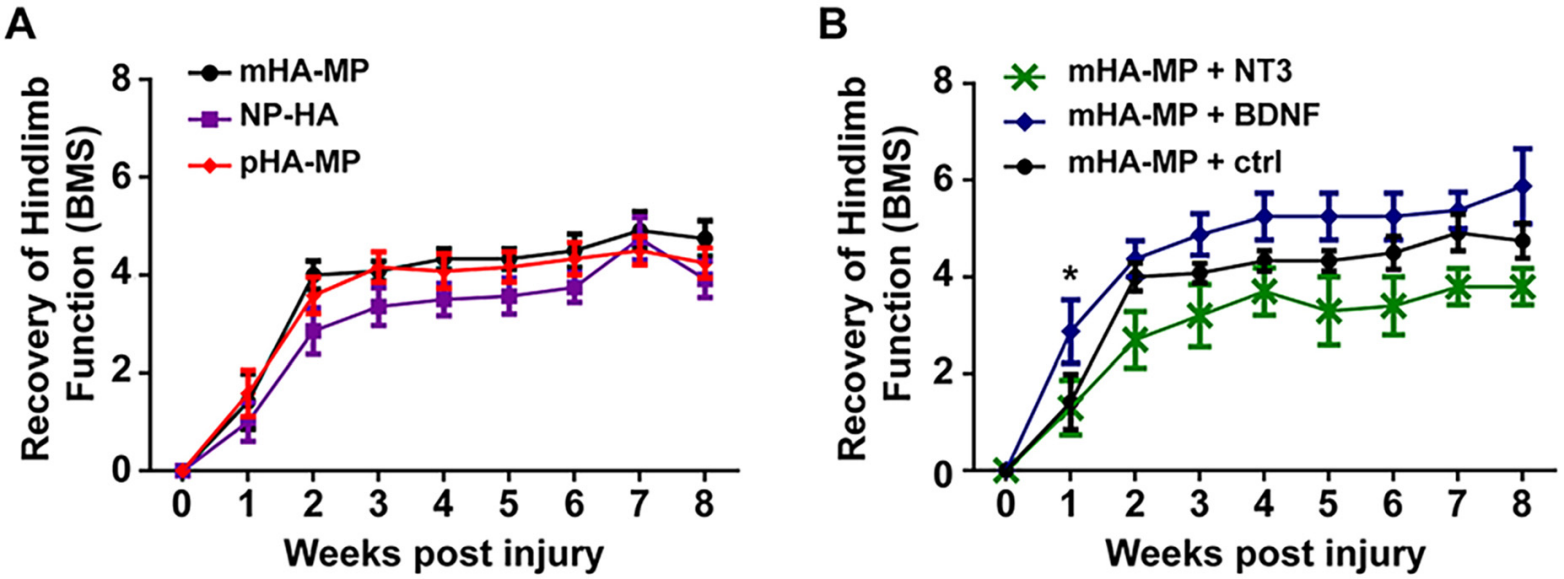
Functional recovery after injury depending on the scaffold type (a) and viral delivery of the BDNF and NT3 within mHA-MP scaffolds (b). Delivery of macroporous scaffolds did not significantly improve regeneration relative to NP-HA scaffolds. The delivery of the BDNF within mHA-MP scaffolds significantly increased regeneration at 1 week post-injury (^*^p <* *0.05, two-way repeated measures ANOVA, Tukey test, n = 4–6).

Functional recovery of hindlimb locomotion (BMS) was evaluated over 8 weeks after SCI. For the most part, there were no significant differences in functional recovery at any time points among animals treated with scaffolds with varying porosities [Fig. 9(a)]. However, when mHA-MP scaffolds were used to deliver lentivirus encoding for BDNF overexpression, there was a non-significant trend toward improved functional recovery over 8 weeks, with a significantly higher BMS score 1 week after SCI [Fig. 9(b)]. This difference in the function at 1 week correlates with increased numbers of myelinated axons in spinal cord lesions at 8 weeks after injury when BDNF transgene was delivered from scaffolds. While not statistically significant, there was an unexpected trend toward worse functional outcomes when NT3 transgene was delivered.

## III. DISCUSSION

After SCI, biomaterial scaffolds can be designed to stabilize the injury site, reduce further tissue damage caused by the secondary injury cascade, and bridge a post-injury microenvironment that inhibits regeneration.^11–13^ Previous studies have shown that networks of cell-sized macropores within biomaterial scaffolds increase infiltration of host cells, improve integration with host tissue, reduce the inflammatory response, and generally support tissue repair *in vivo*.^14,46^ Additionally, macroporosity enables efficient delivery of regenerative factors using genetic vectors.^19,62^ Biomaterials that can be injected and formed *in situ* are particularly attractive as regenerative scaffolds after SCI given irregularly shaped lesions and a need to preserve tissue spared by the primary injury. Here, we investigated how the porous architecture of injectable biomaterials affects their therapeutic benefits after SCI. In addition, we report the ability of scaffolds that are both injectable and macroporous to enhance wound healing and delivery of therapeutic transgenes after SCI.

While delivery from both pHA-MP and mHA-MP scaffolds resulted in increased expression of the FLuc transgene (indicated by bioluminescence intensity measurements) compared to NP-HA (Fig. 1), a corresponding increase in the number of cells that had infiltrated to scaffold centers 2 weeks post-injury was only observed with mHA-MP scaffolds [Fig. 4(c)]. Previous work demonstrated that scaffolds formed from monodisperse, as opposed to polydisperse, microparticles have a greater and more uniform void space that can lead to greater cell proliferation and infiltration *in vivo*.^58^ We posit that this larger, more uniform void space in mHA-MP scaffolds facilitated a more even distribution of cells and fewer cell aggregates compared to other scaffolds [Fig. 4(b)], which could influence the quantification of nuclei. As nuclei that are more spread out are more easily distinguished by CellProfiler software, the presence of tightly packed cell clusters in NP-HA and pHA-MP scaffolds may lead to an underestimation of cell numbers. Although mHA-MP scaffolds had a larger void space than pHA-MP scaffolds [Fig. S1(G)], there were no significant differences in transgene expression or cell infiltration between mHA-MP and pHA-MP scaffolds. While increased numbers of infiltrating cells would be expected to result in an increase in transgene expression,^23,54–56^ it is possible that this effect was not significant enough to elicit a difference in transgene expression (at least at the measurement sensitivity) or that viral dose, which was held constant, was a more significant limiting factor to the numbers of cells transduced.

In contrast to bioluminescence data, histological analysis of td-Tomato transgene expression showed significantly more total expression in mHA-MP scaffolds than in pHA-MP or NP-HA scaffolds [Fig. 2(e)]. Differences in measurement type and the time point (2 weeks after SCI for FLuc and 8 weeks for td-Tomato) could be responsible for this difference. For example, proliferation of transgene-expressing cells may have amplified differences between expression levels at 2 and 8 weeks. While bioluminescence data constitute a cumulative measure of transgene expression, fluorescence intensity in histological sections allows visualization of transgene expression locally in the spinal cord. Research has also shown that the use of scaffolds to deliver gene therapy can improve localization of expression over bolus delivery.^55,62–64^ Our results found no significant effect of macroporosity of the scaffold on localization and distribution of transgene expression in the spinal cord (Fig. 2). Comparing these measures of transgene expression at the tissue level 2 weeks after injury (Fig. 2) and the cell level 8 weeks after injury (Fig. 3) suggests that the distribution of transgene remained primarily localized near the site of delivery across the study duration, in agreement with previous reports.^23,46,47^

While around 1.2-fold more infiltrating cells were observed in both types of macroporous scaffolds 2 weeks after SCI than in nanoporous scaffolds (although the pHA-MP condition was not significantly different), macroporous scaffolds exhibited an over fivefold difference (mHA-MP and pHA-MP each significantly more than NP-HA) in transgene expression as observed by FLuc bioluminescence 2 weeks after SCI (Fig. 1) and roughly twofold difference in transgene expression as assessed by the integrated intensity of td-Tomato fluorescence 8 weeks after SCI (Fig. 2). Together, these data indicate that small changes in numbers of infiltrating cells can lead to large differences in transgene expression. As lentivirus will degrade within a day or two of scaffold implantation, cell infiltration and transduction within this timeframe are imperative.^63,65^ While there were no large differences in cell infiltration among scaffolds with different porosities 2 weeks after implantation, the macroporous architecture of mHA-MP and pHA-MP scaffolds may have encouraged more very early cell infiltration, thus increasing the numbers of cells transduced. However, further investigation at early time points would be required to make any strong claims.

The HA-based scaffolds used in this study are biodegradable, both enzymatically through cell-produced hyaluronidase^12,26,66–68^ and hydrolytically through attack of thioether crosslinks.^69^ Previous reports have observed limited degradation of nanoporous, HA-based scaffolds injected into the injured rodent spinal cord 8–9 weeks after injury.^12,28,66^ In theory, increased cell infiltration into macroporous scaffolds could increase the rate of scaffold degradation, as infiltrating cells may secrete hyaluronidase and affect the local pH. Here, all scaffolds (nanoporous and macroporous) were clearly visible in histological sections 2 weeks after SCI [Fig. 4(a)] but less apparent after 8 weeks [Fig. 4(d)]. While the scaffolds used here were not pre-labeled before implantation and immunohistochemistry against scaffolds was not possible due to the overwhelming presence of endogenous HA, in many tissue sections showing macroporous scaffolds annealed from microparticles, infiltrating axons appear in circular patterns, which may indicate wrapping around a spherical, microparticle surface [Figs. 7(d) and 8(d)]. It is also possible that transduced cells may migrate away from the lesion site as scaffolds degrade. However, this is not likely the case here given that transgene expression remained localized to the lesion area after 8 weeks (Fig. 3), a finding reported by previous studies delivering genetic vectors from degradable biomaterials after SCI.^23,46,47^ Additional studies will be required to characterize the relationships among porous architecture, cell infiltration, degradation rate, and transgene expression patterns in the injured spinal cord.

Areas of F4/80^+^ staining (macrophages and microglia) within scaffolds (Fig. 4) and GFAP^+^ astrocyte staining around scaffolds (Fig. 5) were not significantly different between porosities 2 weeks after injury, suggesting that porosity alone did not significantly affect the inflammatory response by the subacute phase of injury. In acute SCI, disruption of the blood-brain barrier leads to an influx of peripheral monocytes/macrophages, which, along with resident microglia, are activated, targeting any potential sources of infection and producing substantial reactive oxygen and nitrogen species and matrix proteases, which dismantle the spinal cord ECM. Through the subacute stage, astrocytes and perivascular fibroblasts also become activated and remodel the local ECM over time, where a newly deposited ECM typically contributes to glial and fibrotic scar tissues, respectively.^7,70^ In rodents, the production of inflammatory cytokines is typically maximal at around 1 week post-injury, and a peak in F4/80^+^ and GFAP^+^ expression in response to these factors is typically found at 1–2 weeks.^71,72^ Fibroblast deposition of fibronectin, which composes the fibrotic scar, also peaks around this time.^70^ Our findings are consistent with these previous observations. While we did not compare SCI alone to SCI with scaffold implants in this study, a number of previous studies have demonstrated that HA-based scaffolds reduce the presence of macrophages/microglia, activated astrocytes, and ECM deposition in the scar.^12,28^ In this study, HA scaffolds induced a similar inflammatory response as observed in these previous studies.^11,12,28,71^ Together, these previous studies and the current results indicate that while HA scaffolds can reduce the inflammatory response, it is not completely eliminated. This is crucial for tissue regeneration, as a complete lack of an inflammatory response, including scar formation, may actually be detrimental to healing.^8^

Addition of integrin-binding RGD peptides into HA scaffolds provides a means for adhesion of host cells and regenerating axons. Recovery after SCI requires that axons extend either through or around the injury site and reestablish functional synapses. The increased void space in mHA-MP scaffolds was sufficient here to significantly increase the density of axons that had penetrated scaffolds 8 weeks after SCI [Fig. 7(e)]. Myelination of axons in the white matter increases the speed of signal propagation and is necessary for many neurological functions. Thus, effective treatment for spinal cord regeneration may require facilitation of axon extension across the injury site followed by myelination of those axons. Secondary injury can also induce apoptosis of myelinated oligodendrocytes, even while axons remain alive and intact,^73,74^ further emphasizing the need to evaluate axon myelination after SCI. Here, significantly higher densities of myelinated axons were found to have infiltrated scaffolds 8 weeks post-injury [Fig. 7(h)]. However, no significant differences were observed in the percentage of infiltrating axons that were myelinated [Fig. 7(g)], indicating that porosity did not necessarily affect the potential for regenerating axons to be myelinated. It should be noted that the presence of MBP^+^ staining after SCI has been associated with myelin debris.^3^ Here, only NF200^+^ axons closely associated with MBP^+^ myelin were considered to be myelinated axons, making it unlikely that myelin debris was incorporated into data.^3,14,75^ Furthermore, myelin debris has been reported to be clear by 8 weeks post-injury in mice.^76^

The delivery of viral vectors from biomaterial scaffolds has been examined previously in several studies.^6,29,46–48,63^ In particular, Tuinstra *et al.* delivered lentivirus encoding for overexpression of the BDNF and NT3 from poly-lactide-co-glycolide (PLG) scaffolds to a thoracic hemisection SCI in rats.^46^ Our results in mice show comparable improvements in axon density and myelination with delivery of these neurotrophic transgenes. While Tuinstra *et al.* found similar densities of axons myelinated within scaffolds with either NT3 or BDNF delivery, our results show significant benefits of the BDNF but no apparent differences between NT3 conditions and controls (Fig. 8). The orientation of axons within scaffolds appeared random in all scaffold and virus conditions [Figs. 7(f) and 8(f)]. While the orientation of axons along the rostral-caudal axis, like in the normal spinal cord, is preferable, these injectable scaffolds do not provide structural features that can guide axons along this axis.

We observed comparable densities of axons and myelinated axons infiltrating scaffolds with NT3 delivery as another study by Thomas and Seidlits *et al.*, using a C57/Bl mouse thoracic hemisection model, found for NT3 delivery from PLG scaffolds.^47^ We found that densities of myelinated axons when the BDNF was delivered were similar to those reported by Thomas and Seidlits *et al.* for NT3 delivery. Notably, BDNF delivery also increased the proportion of infiltrating axons that were myelinated compared to NT3 or control vector delivery [Fig. 8(g)], indicating an additional benefit of adding BDNF delivery to scaffolds.

Thomas and Seidlits *et al.* found that NT3 delivery primarily increased myelination of axons 8 weeks after SCI by P0^+^ Schwann cells, rather than oligodendrocytes.^47^ In the current study, we observed an increase in the density of P0^+^ Schwann cells in NT3-loaded scaffolds compared to BDNF-loaded scaffolds but not control vector-loaded scaffolds (Fig. S5). However, this difference did not translate into more myelinated axons (Fig. 8). Our data do not show any significant differences between animals treated with vectors encoding NT3 overexpression and those treated with vectors expressing an FLuc control. This unexpected result may be a result of low expression or potency of the NT3 vector relative to the BDNF (Fig. S4) or loss of viral activity during sample preparation. Alternatively, it is possible that suppression of neural stem cell activity by NT3 may have impaired recovery. For example, Delgado *et al.* showed that NT3 can, in some instances, lead to quiescence of neural stem cells through induction of nitric oxide production, which is already elevated after SCI.^77^

Despite significantly greater numbers of axons and myelinated axons 8 weeks post-injury in mHA-MP (Fig. 7), no corresponding benefit in functional recovery was observed over 8 weeks [Fig. 9(a)]. Addition of BDNF transgene delivery significantly increased the density of myelinated axons over mHA-MP scaffolds bearing control FLuc transgene (Fig. 8). This increase translated into a trend toward improved functional recovery over 8 weeks after SCI [Fig. 9(b)]. However, this difference was only statistically significant at 1 week post-injury. Several groups have reported similar results, where the increased axon density or reduced inflammation in response to a particular intervention did not translate into an improved function unless combined with an additional bioactive therapy, including the delivery of the platelet-derived growth factor,^78^ NT3,^42^ NT3 paired with stem cells,^79^ or BDNF.^30^

As the purpose of this study was to evaluate how the pore structure of biomaterial scaffolds affects transgene delivery and regeneration after acute SCI, we did not include an experimental group without a biomaterial implant. Previous studies have evaluated the recovery of C57/Bl6 mice after thoracic SCI compression in the absence of treatment, reporting a maximum BMS score of around 3–4, which peaks 2–4 weeks after injury.^80–82^ In this study, a minimum BMS score of four was found for mHA-MP, pHA-MP, and mHA-MP + BDNF conditions 2 weeks after SCI, and the average BMS score for the mHA-MP + BDNF condition approached six after 8 weeks in the [Fig. 9(b)]. Given this comparison to BMS scores of untreated SCI in other studies^80–82^ and a number of reports demonstrating that HA-based scaffolds can improve functional recovery in rodent SC models, compared to untreated controls,^12,66–68,83^ we expect that the HA-based scaffolds reported here also provide benefits compared to baseline recovery. However, given the variation in BMS scores reported for baseline SCI in the literature across mouse strain, age, sex, injury level, injury severity, and potential subjectivity of the scorer, it is not possible to make a rigorous assessment of how scaffolds may affect recovery over the baseline without including non-treated animals side-by-side in the same study.^80–82,84^

The results described here demonstrate significant potential for the use of the HA-based, injectable, macroporous scaffolds used here in tissue engineering approaches to SCI repair. In particular, these scaffolds represent an effective platform for combined delivery of such therapeutic factors using viral vectors.

## IV. CONCLUSIONS

Tissue engineering strategies for SCI recovery that can reduce the severity of injury while improving the capacity for tissue regeneration are a promising avenue of research. Biomaterial scaffolds can act as inductive platforms for *in situ* tissue repair and deliver additional regenerative therapies, such as genetic vectors. Injectable scaffolds that form *in situ* will likely be required to accommodate variably shaped and sized lesions and maximize tissue sparing. Furthermore, these injectable scaffolds should have cell-scale macropores to support cell and axon infiltration and integrate with host tissue. Here, we demonstrate that scaffolds formed by cross-linking HA-based hydrogel microparticles together *in situ* can fulfill these requirements. The results provide strong evidence that a regularly structured, macroporous network within scaffolds improves the efficiency of transgene delivery and densities of total and myelinated axons infiltrating scaffolds. The delivery of a potentially regenerative transgene, BDNF, further increased densities of total and myelinated axons infiltrating scaffolds, which translated into modest functional recovery. Here, we demonstrate that crosslinked microparticle scaffolds can provide a tissue-inductive platform for combinatorial gene therapies after SCI.

## V. METHODS

Materials were purchased from Fisher scientific unless otherwise specified.

### A. Synthesis and characterization of thiolated hyaluronic acid (HA-SH)

Sodium hyaluronate (M_w_ = 700 kDa, LifeCore Biomedical) was dissolved at 10 mg/mL in distilled, de-ionized water (di H_2_O) and thiolated as previously described.^85^ Molar ratios are reported with respect to carboxyl groups on glucuronic acid moieties of HA. The pH of the HA solution was adjusted to 5.5 using 0.1 M HCl. 1-Ethyl-3-[3-dimethylaminopropyl]carbodiimide (EDC, Fisher Scientific) was dissolved in di H_2_O at a molar ratio of 0.1875 immediately before addition to HA solution. N-hydroxysuccinimide (NHS, Acros Organics) was, then, added at a molar ratio of 0.094. The pH was, then, readjusted to 5.5, and the reaction was mixed at room temperature for 45 min. Then, cystamine dihydrochloride (Sigma-Aldrich) was added (molar ratio, 0.1875), pH was adjusted to 6.25 using 0.1 M NaOH, and the reaction continued while stirring at room temperature overnight. Dithiothreitol (DTT, Sigma-Aldrich) was added in excess (4× greater than cystamine) at pH 8. The mixture was stirred for 1–2 h to cleave cystamine disulfides and yield thiolated HA (HA-SH). The reaction was quenched by adjusting the pH to 4. HA-SH was purified using dialysis against acidic (pH 4) di H_2_O for 3 days in the dark. Purified, HA-SH was filtered through a 0.22 *μ*m filter (EMD Millipore), frozen under liquid nitrogen, lyophilized, and stored at −20 °C until use. HA thiolation was confirmed using proton nuclear magnetic resonance (NMR) spectroscopy and the colorimetric Ellman test for free thiols.^86^

### B. Gene therapy design

Lentiviral particles encoding firefly luciferase (FLuc), td-Tomato, BDNF, or NT3, each under control of a constitutively active cytomegalovirus (CMV) promoter, were generated using a third generation packaging system, as previously described.^87^ Plasmids were generously provided by Professor Lonnie Shea at the University of Michigan.^46–48^ Briefly, 80% confluent human embryonic kidney (HEK) cells (Lenti-X 293T Takara Bio, USA) were simultaneously transfected with packaging plasmids using jetPRIME transfection reagent (Polyplus transfection)—pCMV-VSV-G (gift from Bob Weinberg, Addgene plasmid # 8454) and pRSV-Rev and pMDLg/pRRE (gifts from Didier Trono; Addgene plasmids # 12253 and #12251, respectively). Lentiviral particles were recovered from media after 2 days of culture using PEG-it virus precipitation solution (SBI System Biosciences), resuspended in D-phosphate-buffered saline (PBS), and stored at −80 °C. Lentiviral titers were calculated using the Lenti-X qRT-PCR titration kit (Takara Bio, USA).

### C. Formation of nanoporous scaffolds (NP-HA)

HA-SH and four-arm vinyl sulfone-terminated polyethylene glycol-vector scan (PEG-VS) (20 kDa, Laysan Bio) were crosslinked via Michael-type addition between thiol and vinyl sulfone functional groups.^88^ HA-SH and PEG-VS were dissolved separately in PBS at pH 7.4. The cysteine-terminated RGD peptide (GCGYGRGDSPG, GenScript Biotech) was conjugated to PEG-VS by reaction at room temperature for 1 h prior to gel formation to provide sites for cell adhesion.^89^ To form hydrogels, precursor solutions of PEG-VS and HA-SH were mixed to yield a mixture with a ratio of 1.2:1 of thiol:vinyl sulfone.^90^ Hydrogels had final concentrations of 10 mg/mL HA-SH, 150 *μ*M peptide, and 6 mg/mL PEG-VS. For *in vitro* studies, HA-SH and peptide-modified PEG-VS solutions were mixed and pipetted into circular wells of a silicone isolator (8 mm diameter, 1 mm depth, Grace BioLabs).^91^ Scaffolds were incubated at 37 °C for 2 h to ensure that cross-linking had completed.^88^ For some *in vitro* materials characterization studies, L-cysteine was conjugated instead of RGD.

### D. Hydrogel microparticle formation

HA-SH was crosslinked with peptide-modified PEG-VS as described above for NP-HA scaffolds. Microparticles were made from NP-HA hydrogels using two methods: (1) batch fabrication using water/oil emulsification, which yields a wide range of particle sizes,^54^ or a step-emulsification microfluidic device, which more tightly controls the microparticle diameter.^57^ To produce polydisperse microparticles by emulsion, 100 *μ*L of hydrogel precursor solution was vortexed in 900 *μ*L mineral oil with 1% span 80 surfactant for 20 s before addition of 100 *μ*L mineral oil containing 0.1% triethylamine (Sigma-Aldrich), an oil-soluble base that raised the precursor pH and initiated cross-linking. The emulsion was vortexed for an additional 20 s and stirred at room temperature overnight in the dark to ensure that cross-linking was complete. Microparticles were centrifuged and washed with mineral oil five times and hexane five times before resuspending in 70% ethanol. Microparticles were, then, sieved twice against 70-*μ*m cell strainers and stored at 4 °C in 70% ethanol. To produce monodisperse microparticles, a microfluidic step-emulsification approach was used as previously described.^57^ Briefly, the precursor solution was co-injected into a 200 channel step-emulsification device along with an oil phase composed of Novec 7500^TM^ + 1% PicoSurf (Sphere Fluidics) to generate monodisperse droplets. After droplet generation, an additional oil phase composed of Novec 7500^TM^ and 3% (vol/vol) triethylamine was introduced in flow to increase precursor pH and facilitate particle cross-linking. The microparticles were recovered from the oil phase using a series of Novec and hexane washing steps before resuspending in 70% ethanol for sterilization and storage. Microparticles were washed three times with sterile PBS immediately before use.

### E. Macroporous scaffold formation and characterization

Microparticle diameters were manually measured using ImageJ (NIH) using at least 500 microparticles of each type across three batches to estimate size distributions [Figs. S1(A)–S1(C)]. Macroporous scaffolds were formed by annealing either monodisperse (mHA-MP) or polydisperse (pHA-MP) microparticles via disulfide bonding, as previously described.^54^ When injected *in* vivo, ethanol-sterilized and PBS-rinsed microparticles or NP-HA precursor was mixed with poly-L-lysine (PLL, Sigma-Aldrich), to facilitate the retention of lentiviral particles, to a final concentration of 100 ng/mL.^55^ Lentiviral solution was added to achieve a final viral dose of 10^6^ to 10^7^ viral particles per scaffold. When produced *in vitro* for physical characterization, PLL and lentiviral solutions were substituted with PBS.

### F. Confocal microscopy to evaluate the scaffold macrostructure

To visualize scaffolds fluorescently, Texas red- or fluorescein-maleimide was conjugated to thiols on HA-SH and incorporated into hydrogels at 0.1 mg/mL. To assess pore interconnectivity, scaffolds were incubated in a 1 mg/mL solution of high molecular weight (500 kDa) fluorescein isothiocyanate (FITC)-dextran (TdB Consultancy AB). FITC-dextran was incubated with Texas-red tagged NP-HA scaffolds to confirm the inability to diffuse into nanoporous scaffolds [Figs. S1(D)–S1(F)]. Scaffolds were imaged using an SP5 confocal microscope to estimate void space (Leica Microsystems). 3D reconstructions were created using the volume viewer plugin for ImageJ (NIH). The void space was calculated using thresholding images using an Otsu algorithm and by calculating the percent area covered by FITC-dextran throughout the scaffold volume [Fig. S1(G)].^92^

### G. Compression thoracic injury model in female mice

All *in vivo* studies were conducted in compliance with the NIH Guide for Care and Use of Laboratory Animals with approval from the UCLA Institutional Animal Care and Use Committee (Protocol #2015–006). Studies were performed on 8–10 weeks old healthy female C57BL6 mice (Charles River, N = 6) with *ad libitum* access to food and water. Pre-operatively, mice were acclimatized to handling and functional assessment procedures for 10 days prior to the procedure. Mice were anesthetized using vaporized isoflurane (3%–4% for induction and 1.5%–3% for maintenance). The spinal cord was exposed by laminectomy (T8-T10), and a clip compression injury using a 30 g, 1 mm microvascular clip (RS-6470, Roboz Surgical) was made at spinal cord level T9 for 15 s.^93,94^ Injections were performed using a 34 G needle (80 *μ*m inner diameter). The animal received an injection to form either NP-HA, pHA-MP, or mHA-MP scaffolds *in situ* (N = 6) immediately after compression injury. At a rate of 1 *μ*L/min, 1 *μ*L of material was injected into the lesion epicenter through a 34 G needle and a 10 *μ*l NanoFil syringe and using a UMP3–1 micropump (World Precision Instruments). After injection, the laminectomy site was covered with Gelfoam (Pfizer) to discourage direct adhesion of muscles to spinal cord tissue. The muscle and subcutaneous tissue were sutured with absorbable 4–0 chromic gut sutures (003–2482, Ace Surgical), and skin was closed using wound clips (427631, BD). Post-operative care included injection with buprenorphine (twice daily) and lactated Ringer's solution (once daily) for 72 h, and bladders were expressed twice daily until normal bladder expression returned.

### H. *In vivo* bioluminescence

Luciferase bioluminescence was measured 2 weeks post-injection of scaffolds loaded with FLuc-encoding lentivirus using an IVIS Lumina II imager (Perkin Elmer) using standard techniques.^54^ Animals were injected with 150 mg/kg of luciferin into the intraperitoneal space, and images were taken every 3 min for 50 min post-injection. Images with the greatest photon flux were used for analysis.

### I. Tissue immunostaining and analysis

Cryosectioning and hematoxylin and eosin (H&E) staining were performed by the Translational Pathology Core Laboratory at the UCLA. Explanted tissues were cryosectioned in the sagittal plane at a thickness of 18 *μ*m. Immunostaining was performed to detect td-Tomato transgene (1:200, LS-C340696, LifeSpan BioSciences, Inc.), F4/80^+^ macrophages/microglia (1:200, MCA497R, AbD Serotec), NF200^+^ axons (1:50, N4142, Sigma-Aldrich), GFAP^+^ astrocytes (1:200, GFAP, Aves Labs), MBP^+^ oligodendrocytes (1:200, SC-13914, Santa Cruz Biotechnology), and P0^+^ Schwann cells (1:200, PZ0, Aves Labs). Secondary antibodies against rat (1:1000, SABt4600133, donkey anti-rat, Sigma-Aldrich) or goat (1:1000, SAB4600032, donkey anti-goat, Sigma-Aldrich) were used, as appropriate. Hoechst 33342 was used as a nuclear counterstain. Wide-field fluorescence images were taken using an Axio-Observer microscope (Carl Zeiss) at 200× magnification with a numerical aperture of 0.8. Staining and imaging were performed in a single batch and using matched exposure levels to enable head-to-head comparisons.

At least two sections with clearly defined injury regions and a visible scaffold from each of at least three animals per condition were used for analysis in all cases. Quantitative analysis of neurofilament (NF200^+^) and myelination (NF200^+^/MBP^+^) present was performed using a modified axon counting and spinal cord regeneration algorithm using a Hessian filtering-based method to improve threshold detection and quantification and the MATLAB code, as previously described.^75^ Calculation of cell densities and integrated intensity, determination of positively stained regions and the angle of the NF200+ object orientation, and quantification of overlapping cell markers were performed using CellProfiler software.^95^ For quantification of td-Tomato integrated intensity over the length of the spinal cord, the integrated intensity was calculated within 300 *μ*m segments from 0 to 1.5 mm away from the center of the scaffold in both longitudinal directions. The percent integrated intensity was calculated by normalizing the intensity within each bin to the total intensity within all bins. Comparison of anti-td-Tomato immunostained and natively fluorescent td-Tomato was performed by comparing thresholding images for the positively stained area using CellProfiler software.^71^

### J. Analysis of functional recovery

Functional recovery after SCI was assessed using the basso mouse scale (BMS) for the locomotor function.^84^ Testing was done prior to injury and weekly after SCI and scaffold injection until mice were euthanized. Mice were placed individually in an open field for 4 min, and hind-limb movements were assessed in accordance with the BMS.

### K. BDNF and NT3 activity

To confirm the activity of the BDNF and NT3 proteins, conditioned media from BDNF- or NT3-overexpressing Lenti-X 293T cells (Takara Bio) was delivered to embryonic (E18) mouse dorsal root ganglia (DRGs, C57EDRG, BrainBits). DRGs were seeded on PLL-coated, glass coverslips in NbActiv4 media containing Neurobasal/B27 and Glutamax supplements (Nb4, BrainBits). After one day of culture, half of the culture media was replaced with conditioned media from HEK cells overexpressing the BDNF, NT3, or FLuc (negative control). DRGs were cultured for 3 days prior to fixation by 4% paraformaldehyde for 10 min and immunocytofluorescence staining and imaging of NF200 with a Hoechst 33342 nuclear counterstain [Figs. S2(A)–S2(C)]. Neurite extensions (NF200^+^) from the DRG body were analyzed using Sholl analysis and quantification of size and density of positive neurites in concentric rings from the center of the DRG.^96^

NT3 production by cells infected with lentivirus was further confirmed by immunoblotting. After infection with NT3-encoding lentiviral particles, HEK cells were cultured for 4 days. The cell medium was collected before cells were lysed by 30-min incubation in RIPA buffer, on ice, followed by 15 min of centrifugation at 14 000× g. The medium and cell lysate were spotted on a nitrocellulose membrane, incubated in 10% BSA blocking solution, and the presence of transgene was detected using the rabbit anti-NT3 antibody (500-P84–50ug, PeproTech) followed by the anti-rabbit HRP-linked secondary antibody (7074S, Cell Signaling Technologies).

### L. Statistical analyses

Data were analyzed using GraphPad Prism six software. Differences in cell counts and immunostaining of cell types were analyzed using the Kruskal-Wallis test followed by Dunn's multiple comparisons test. Data are displayed as the mean ± standard error of the mean with significance considered to be *p *<* *0.05. The recovery of the hindlimb function (BMS) was analyzed via two-way ANOVA, where independent variables were time and scaffold type. Differences in FLuc expression were evaluated using a nonparametric bootstrapping approach.^97^ Two-sided 95% confidence intervals were calculated with 10 000 iterations using MATLAB software loaded with the Statistics and Machine Learning Toolbox. Significance denotes no overlap between 95% confidence intervals.

## SUPPLEMENTARY MATERIAL

See the supplementary material for Figs. S1–S5.

## AUTHORS' CONTRIBUTIONS

S.K.S. conceptualized the study; S.K.S., A.E., and D.D. designed the study; A.E., M.S., L.M.R., J.dR., R.D.B., J.L., and W.X. performed laboratory investigation; A.E., J.dR., and M.S. analyzed data; A.E. and S.K.S. wrote the manuscript; A.E., L.M.R., R.D.B., J.L., and S.K.S. reviewed and edited the manuscript; and S.K.S. and D.D. supervised and provided resources. All authors had substantial input to the logistics of the work and approved the final manuscript.

## Data Availability

The data that support the findings of this study are available within the article and its supplementary material. Additional raw data that support the findings of this study are available from the corresponding author upon reasonable request.

## NOMENCLATURE

BDNF: brain-derived neurotrophic factor
FLuc: firefly luciferase
HA: hyaluronic acid
NT3: neurotrophin 3
